# Supplementary material for: SARS-CoV-2 and its ORF3a, E and M viroporins activate inflammasome in human macrophages and induce of IL-1α in pulmonary epithelial and endothelial cells
Source: Cell Death Discov. 2024 Apr 25;10:191. doi: 10.1038/s41420-024-01966-9 (PMC11045860; doi:10.1038/s41420-024-01966-9)

SARS-CoV-2 and its ORF3a, E and M viroporins activate inflammasome in human macrophages and induce of IL-1α in pulmonary epithelial and endothelial cells

Magdalena Ambrożek-Latecka[1], Piotr Kozlowski[2], Grażyna Hoser[1], Magdalena Bandyszewska[1], Karolina Hanusek[3], Dominika Nowis[4], Jakub Gołąb[4], Małgorzata Grzanka[3], Agnieszka Piekiełko-Witkowska[3], Luise Schulz[5], Franziska Hornung[5], Stefanie Deinhardt-Emmer[5], Ewa Kozlowska[6], Tomasz Skirecki[1*]

Supplementary results

Supplementary Figure 1. NLRP3 inflammasome is engaged in IL-*β* and gasdermin D maturation upon SARS-CoV-2 viroporin expression. Densitometric analysis of Western blots from U937 WT and U937 NLRP3 KO cells transfected with lentiviral particles carrying ORF3A, E and M proteins. A. IL-1 *β*. B. Gasdermin D (GSDMD). Graphs represent means ± SEM, n=3. Multiple comparisons were performed with two-way ANOVA test. ****p<0.0001.

A

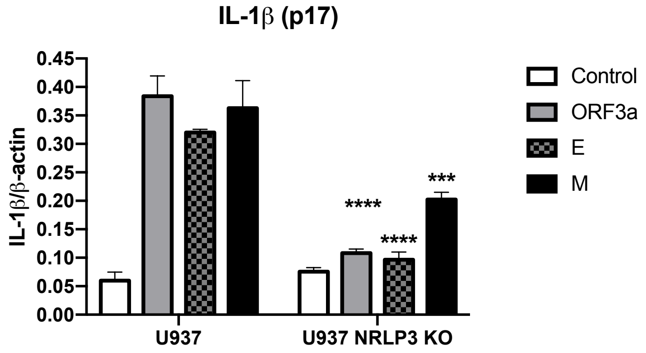

B

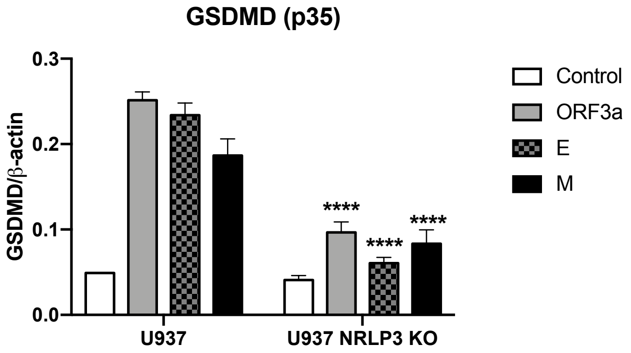

Supplementary Figure 2. Validation of the novel inflammasome reporter cell lines. Immortalized cell lines expressing the ASC::GFP protein were stimulated with LPS and nigericin. A. human bronchial epithelial cells -3 KT cell line. B. human umbilical vein endothelial cells/TERT cell line.

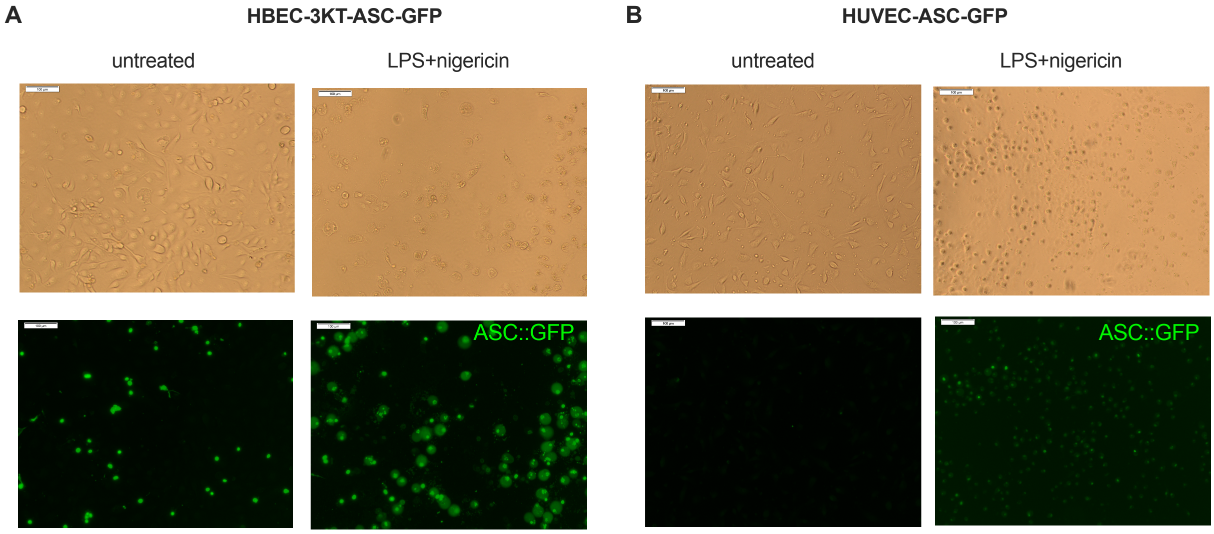

Supplementary Figure 3. Analysis of the activation of apoptotic caspases in human pulmonary epithelial cells and human pulmonary microvascular endothelium by the viroporins A. Activation of caspase-3/7 in HPAEpiCs by the viroporins; representative pictures (left) and fluorometric readouts (right) are shown. F. Activation of caspase-3/7 in HPMECs by the viroporins; representative pictures (left) and fluorometric readouts (right) are shown. Graphs represent means ± SEM, n=3-4. Two groups comparisons were analyzed with t-test. Multiple comparisons were performed with two-way ANOVA test. *p<0.05.

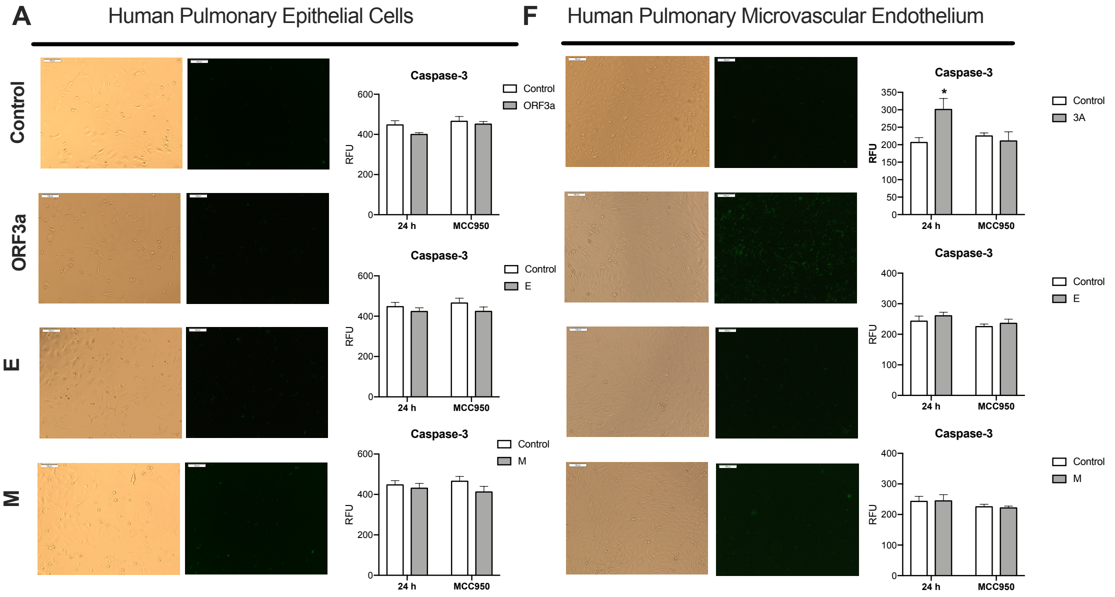

Supplement: Supplementary file 1 — Supplementary Material [file 41420_2024_1966_MOESM1_ESM.pdf]
