# Supplementary material for: SARS-CoV-2 and its ORF3a, E and M viroporins activate inflammasome in human macrophages and induce of IL-1α in pulmonary epithelial and endothelial cells
Source: Cell Death Discov. 2024 Apr 25;10:191. doi: 10.1038/s41420-024-01966-9 (PMC11045860; doi:10.1038/s41420-024-01966-9)
Supplement: Supplementary file 2 — Supplementary Material WB [file 41420_2024_1966_MOESM2_ESM.docx]

**Supplementary Figure 4. Uncropped immunoblots of the different figures.**
